# Hybrid PET/MR Kernelised Expectation Maximisation Reconstruction for Improved Image-Derived Estimation of the Input Function from the Aorta of Rabbits

**DOI:** 10.1155/2019/3438093

**Published:** 2019-01-16

**Authors:** Daniel Deidda, Nicolas A. Karakatsanis, Philip M. Robson, Claudia Calcagno, Max L. Senders, Willem J. M. Mulder, Zahi A. Fayad, Robert G. Aykroyd, Charalampos Tsoumpas

**Affiliations:** ^1^Biomedical Imaging Science Department, University of Leeds, Leeds, UK; ^2^Department of Statistics, University of Leeds, Leeds, UK; ^3^Translational and Molecular Imaging Institute (TMII), Department of Radiology, Icahn School of Medicine at Mount Sinai, New York, NY, USA; ^4^Division of Radiopharmaceutical Sciences, Department of Radiology, Weill Cornell Medical College, Cornell University, New York, NY, USA

## Abstract

Positron emission tomography (PET) provides simple noninvasive imaging biomarkers for multiple human diseases which can be used to produce quantitative information from single static images or to monitor dynamic processes. Such kinetic studies often require the tracer input function (IF) to be measured but, in contrast to direct blood sampling, the image-derived input function (IDIF) provides a noninvasive alternative technique to estimate the IF. Accurate estimation can, in general, be challenging due to the partial volume effect (PVE), which is particularly important in preclinical work on small animals. The recently proposed hybrid kernelised ordered subsets expectation maximisation (HKEM) method has been shown to improve accuracy and contrast across a range of different datasets and count levels and can be used on PET/MR or PET/CT data. In this work, we apply the method with the purpose of providing accurate estimates of the aorta IDIF for rabbit PET studies. In addition, we proposed a method for the extraction of the aorta region of interest (ROI) using the MR and the HKEM image, to minimise the PVE within the rabbit aortic region—a method which can be directly transferred to the clinical setting. A realistic simulation study was performed with ten independent noise realisations while two, real data, rabbit datasets, acquired with the Biograph Siemens mMR PET/MR scanner, were also considered. For reference and comparison, the data were reconstructed using OSEM, OSEM with Gaussian postfilter and KEM, as well as HKEM. The results across the simulated datasets and different time frames show reduced PVE and accurate IDIF values for the proposed method, with 5% average bias (0.8% minimum and 16% maximum bias). Consistent results were obtained with the real datasets. The results of this study demonstrate that HKEM can be used to accurately estimate the IDIF in preclinical PET/MR studies, such as rabbit mMR data, as well as in clinical human studies. The proposed algorithm is made available as part of an open software library, and it can be used equally successfully on human or animal data acquired from a variety of PET/MR or PET/CT scanners.

## 1. Introduction

[^18^F]-based PET imaging has been successfully used as a noninvasive imaging biomarker of different human diseases. [^18^F]-Sodium fluoride ([^18^F]-NaF) has been associated with calcium molecular metabolism, and it has been used to study benign osseous diseases such as osteoporosis, vascular calcification, osteoarthritis, and rheumatoid arthritis [1–6]. [^18^F]-Fluodeoxyglucose ([^18^F]-FDG) is the most commonly used in clinical practice and particularly for the detection, quantification, staging, and therapy evaluation of cancerous lesions, as well as in cardiovascular and neurological diseases [7–12].

Accurate and precise quantitative biomarkers can be obtained by exploiting the pharmacokinetic information in the measured data [13]. This requires the estimation of the radiotracer concentration in the arterial blood plasma (input function). The gold standard for such measurement is blood sampling during the PET acquisition, via arterial cannulation [14]. Unfortunately, this technique is invasive and can be complicated, as it requires arterial blood samples in specific quantities and at precise times with corrections for delay and dispersion to account for the distance between the sampling site and the regions of interest (ROIs) [15].

A noninvasive technique is the image-derived input function (IDIF) [16] which uses a region of interest (ROI) to measure the uptake in the vessel over time. The IDIF is a simple way to calculate activity over time; however, it is challenging due to image-related issues. Firstly, the choice of the ROI has a very important impact, and nonaccurate ROIs will affect the measurement [17, 18]. Other challenges are related to the use of MR images to extract the ROI because a potentially inaccurate registration between PET and MR images can lead to erroneous estimates of the activity in the chosen arterial ROI. With a hybrid PET/MR scanner, the problem of coregistration is expected to be minimised.

The aforementioned problems are mostly related to the ordered subsets expectation maximisation (OSEM) method [19] which is usually followed by postreconstruction Gaussian filtering due to the high noise levels expected for the very short-time frames used for the IDIF estimation. OSEM with or without postfiltering has been shown to produce inaccurate values of IDIF with bias up to 30% propagating through the kinetic constant calculations [20]. In preclinical experiments, these issues can be even more challenging [13, 21] because of the smaller size of animal vessel tissue, such as rabbit aortas, especially when they are performed with clinical scanners designed for larger human subjects. In this case, the PVE can be significant, as the diameter of the rabbit aorta is about 5 mm which is the same order of magnitude as the PET resolution.

Different studies have proposed methods for the use of IDIF by correcting or avoiding PVE [22–26]. Zanotti-Fregonella et al. [16] have shown in their comparison between cannulation-based and image-derived input functions that the use of high-resolution PET images is often not sufficient to avoid the need of cannulation to obtain a reliable IDIF. Moreover, the accuracy of the IDIF may vary between radiotracers and scanners. MR-guided techniques have been proposed and discussed [15], showing that erroneous registration between the PET and the MR images, as well as erroneous MR segmentation, can introduce an error in the IDIF estimation. The problem of PET/MR misalignment has been discussed for the kernel by Deidda et al. [27]. In this study, we apply a PET/MR-guided image reconstruction algorithm, hybrid kernelised expectation maximisation (HKEM) [28], to minimise PVE during the reconstruction step so that we can obtain more accurate IDIF estimates. In addition, to minimise the PET/MR misalignment, the HKEM-reconstructed image at the peak activity frame was used together with the MR image to extract the ROI to be used for the estimation of the input function. In this way, only a percentage of the maximum value is included in the ROI avoiding low-value voxels outside the carotid in case of PET/MR misalignment.

The kernel method [29], which was first introduced in PET image reconstruction by Wang and Qi [30] and Hutchcroft et al. [31, 32], makes use of only one prior information image, MR or PET, respectively. Furthermore, many other studies showing promising performances have appeared in the literature [33–39]. In contrast, the HKEM method, which we recently developed in the open-source STIR library [40], exploits both the PET and the MR coregistered images to derive PET information iteration after iteration.

The HKEM algorithm was introduced by Deidda et al. [28] as a method for improving PET image resolution and uptake recovery in PET/MR phantom experiments, as well as contrast and quantification of atherosclerotic plaque lesions in carotid arteries in clinical PET/MR studies—which could also be applied in PET/CT studies. In addition, it is a robust and stable method which gives consistent results across different datasets using the same parameter settings. In this paper, we focus on the quantification of the aorta IDIF of rabbits using ^18^F-based radiotracers such as [^18^F]-FDG and [^18^F]-NaF, to extend the applicability and usefulness of our novel reconstruction algorithm. Here, we assume that if HKEM can recover the uptake while retaining satisfactory noise suppression for low-count PET acquisitions, it will also be capable of providing accurate IDIF estimates using a wide range of dynamic PET frame durations.

The paper is structured as follows: Section 2 describes the datasets used to study image reconstruction, list mode (LM) subsampling, and the experimental methodology.  Section 3 presents the results of the proposed method and comparison with different standard algorithms. The results are discussed in Section 4, and conclusions are drawn in Section 5.

## 2. Methods and Materials

### 2.1. Simulation

A realistic simulation was created using a model derived from real [^18^F]-NaF rabbit data [41] and utilities implemented in the STIR library. The real data were acquired with the Siemens Biograph mMR scanner at Mount Sinai Hospital, NY, USA. The voxel size for the simulated image was 1.56 × 1.56 × 2.031 mm. The rabbit was a healthy subject and was anaesthetised before the scan. It was injected with [^18^F]-NaF 170 MBq and scanned for 90 minutes. Different organs and tissues were segmented from the acquired MR UTE sequence, using 0.07 ms echo time. The original voxel size is 1.56 × 1.56 × 1.56 mm. It is then aligned to the PET field of view (FOV) and resliced to match the PET native voxel size, 1.56 × 1.56 × 2.031 mm^3^, and FOV size, 344 × 344 × 127 voxels. The same image is also used for the calculation of the kernel matrix. In particular, the abdominal aorta, kidneys, bladder, myocardium, lungs, stomach, and background were extracted as independent images. Each tissue type was segmented using a semiautomatic segmentation method in ITK-SNAP based on thresholding [39], and it was then used as a ROI in the real PET data to estimate the activity concentration over 45 frames organised as follows: 17 × 6 s, 4 × 15 s, 4 × 30 s, 4 × 60 s, 4 × 180 s, and 12 × 300 s. The measured values were then assigned to every tissue in the simulation.

In order to create the projection data, each simulated image is forward projected into the sinogram space. The attenuation sinogram is estimated using the attenuation coefficient, *μ*, map obtained from a Dixon MR sequence [42–45], and the precalculated hardware attenuation coefficients for the bed and coils. The projection data containing random events were estimated as a uniform sinogram containing 20% of the total number of events in the simulated acquisition sinogram. In order to estimate the scattered events, the Watson single scatter simulation was applied [46], and a mask obtained from the *μ* map was used for the tail fitting. At this point, the random and scatter sinograms were combined as an additive term in the emission sinogram to create the modelled prompts projection data. The final step was the simulation of Poisson noise from the prompts events.

The above steps were repeated for each simulated frame image, and 10 noise realisations were created.

### 2.2. Real Rabbit Data

The acquisition was carried out using the Siemens Biograph mMR at Mount Sinai Hospital, NY, USA. The rabbit was a healthy subject and was anaesthetised during the scan. It was injected with [^18^F]-NaF 170 MBq for the first study and [^18^F]-FDG 133 MBq for the second, both scanned for 90 minutes. The attenuation images were obtained from the scanner, which included the attenuation coefficient for bed and coils. The LM data were divided into smaller frames, to permit calculation of the input function. The tracer was injected during the first seconds of the scan. The MR part of the kernel matrix was obtained from a MR UTE sequence with 0.07 ms echo time, and the original voxel size was 1.56 × 1.56 × 1.56 mm. It was then aligned to the PET field of view (FOV) and resliced to match the PET native z voxel size, 1.56 × 1.56 × 2.031 mm^3^, and FOV size, 344 × 344 × 127 voxels.

### 2.3. Reconstruction Setup

All the datasets were reconstructed using HKEM with 21 subsets and 10 iterations. The PET image voxel, *λ*_*j*_, using the HKEM can be written as

(1) $$\begin{array}{c} \lambda_{j}=\overset{N_{j}}{\underset{f=1}{\sum }}\alpha_{f}k_{fj}, \end{array}$$

where *k*_*fj*_ is the *fj* ^*th*^ element of the kernel, *N*_*j*_ is the number of feature vectors related to voxel *j*, and *α*_*f*_ is the kernel coefficient to be estimated iteratively as follows:

(2) $$\begin{array}{c} \alpha_{f}^{\left(n+1\right)}=\frac{\alpha_{f}^{\left(n\right)}}{\sum_{j}k_{fj}^{\left(n\right)}\sum_{i}p_{fi}}\underset{j}{\sum }k_{fj}^{\left(n\right)}\underset{i}{\sum }p_{ij}\frac{1}{\sum_{l}p_{il}\sum_{f}k_{fl}^{\left(n\right)}\alpha_{f}^{\left(n\right)}+s_{i}}, \end{array}$$

with *p*_*ij*_ being the system matrix and *s*_*i*_ the additive term. The *fj* ^*th*^ element of the kernel consists in two components, and it can be written as follows:

(3) $$\begin{array}{c} k_{fj}^{\left(n\right)}=k_{m}\left(v_{f},v_{j}\right)\cdot k_{p}\left(z_{f}^{\left(n\right)},z_{j}^{\left(n\right)}\right), \end{array}$$

where

(4) $$\begin{array}{c} k_{m}\left(v_{f},v_{j}\right)=\exp\left(-\frac{{\left|\left|v_{f}-v_{j}\right|\right|}^{2}}{2\sigma_{m}^{2}}\right)\exp\left(-\frac{{\left|\left|x_{f}-x_{j}\right|\right|}^{2}}{2\sigma_{dm}^{2}}\right), \end{array}$$

is the kernel derived from the MR image and

(5) $$\begin{array}{c} k_{p}\left(z_{f}^{\left(n\right)},z_{j}^{\left(n\right)}\right)=\exp\left(-\frac{{\left|\left|z_{f}^{\left(n\right)}-z_{j}^{\left(n\right)}\right|\right|}^{2}}{2\sigma_{p}^{2}}\right)\exp\left(-\frac{{\left|\left|x_{f}-x_{j}\right|\right|}^{2}}{2\sigma_{dp}^{2}}\right), \end{array}$$

is the kernel component derived from the updated PET image. The Gaussian kernel functions have been modulated by the distance between voxels in the image space. The quantity **x**_*j*_ is the coordinate of the *j* ^*th*^ voxel, *n* is the subiteration number, **z**_*j*_^(*n*)^ and **v**_*j*_ are the feature vectors that are calculated from the *n* ^*th*^ updated PET image and the MR image, respectively, and *σ*_*m*_, *σ*_*p*_, *σ*_*dm*_, and *σ*_*dp*_ are the scaling parameters for the distances in (4) and (5). Note that the HKEM uses a voxel-wise kernel. This means that the feature vector assigned for each voxel contains only one nonzero element with the same voxel value.

The kernel parameters were chosen in order to obtain the minimum RMSE in the aorta. The values of the kernel parameters were set as follows: *N*=27, *σ*_*m*_ = 1, *σ*_*dm*_ = 3, *σ*_*p*_=1, and *σ*_*dp*_ = 3 (the last two are only used by HKEM).

For comparison, the same datasets have been reconstructed also with 21 subsets and 10 iterations of OSEM with and without 3 mm FWHM Gaussian postfilter. These methods are denoted as OSEM+G and OSEM, respectively, in this study. The selected number of subsets and the application of the Gaussian post-filter are considered as standard settings in clinical routine. All datasets were reconstructed using span 1.

Scatter correction was performed with the method described by Tsoumpas et al. [47] and Polycarpou et al. [48]. Randoms were estimated from singles, which were calculated from delayed events [49]. The procedures for these evaluations, including attenuation and normalisation corrections [50], make use of STIR.

### 2.4. Image Analysis

**Figure 1:**
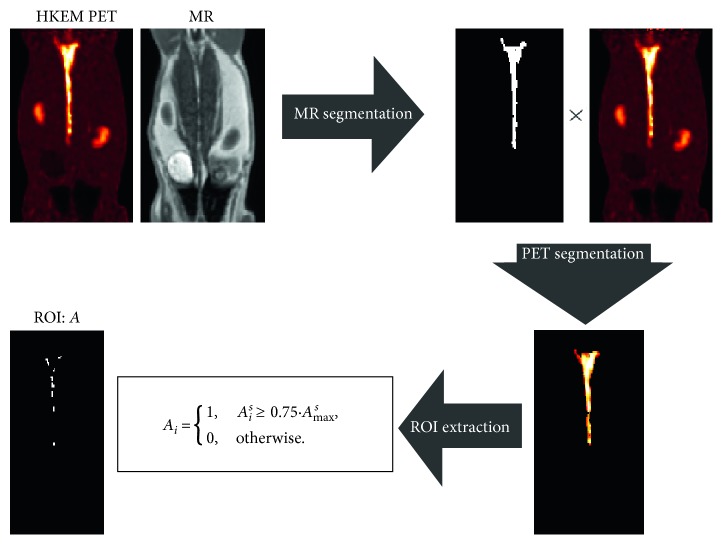
Schematic representation of the extraction of the region of interest (ROI), *A*, of the aorta using the PET and MR images as the input.

**Figure 2:**
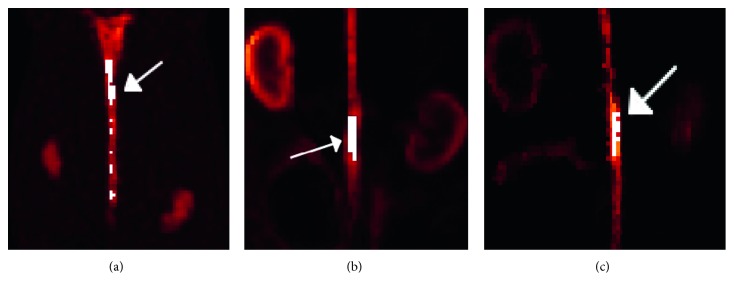
Regions of interest (ROIs) chosen for this study, defined by the white regions. The target ROIs for the (a) aorta in the simulation, (b) [^18^F]-NaF rabbit study, and (c) [^18^F]-FDG rabbit study. The target ROIs are indicated by the white arrows.

The comparison was carried out in terms of the mean value for all of the short frames and datasets, and the bias was estimated for the simulation to assess the accuracy of the proposed method. The ROI was obtained using the HKEM-reconstructed image and the MR image as follows (Figure 1):

- The aorta was segmented from the MR image using the semiautomatic segmentation method in ITK-SNAP based on thresholding [51]
- The obtained mask is multiplied with the HKEM-reconstructed PET image to obtain the segmented aorta, *A* ^*s*^, from the the PET image
- The ROI, *A*, is obtained by taking into account only the voxels with a value bigger than 75% of the maximum in order to optimize those affected by PVE

(6) $$\begin{array}{c} A_{i}=\left\{\begin{array}{c} 1,\;A_{i}^{s}\geq 0.75\cdot A_{max}^{s},\\ 0,\;\text{otherwise}, \end{array}\right. \end{array}$$

where *I* is the index of the voxel. Quantitative comparison between algorithms was performed using the following figures of merit:

(7) $$\begin{array}{c} {\text{mean}}_{k}=t_{k}=\frac{{\sum^{}}_{j=1}^{V}t_{jk}}{V}, \end{array}$$

(8) $$\begin{array}{c} {\text{bias}}_{k}=\frac{\left|t_{k}-A_{k}^{T}\right|}{A_{k}^{T}}\cdot 100, \end{array}$$

(9) $$\begin{array}{c} {\text{CoV}}_{k}=\frac{\sqrt{1/\left(V-1\right){\sum^{}}_{j=1}^{V}{\left(t_{jk}-t_{k}\right)}^{2}}}{t_{k}}\times 100, \end{array}$$

where *t*_*k*_ is the mean value of the target ROI at frame *k*, *t*_*jk*_ is the value of voxel *j* within the ROI at frame *k*, and *V* is the number of voxels within the ROI. The ROIs obtained with the proposed method are shown for each dataset in Figure 2.

## 3. Results

### 3.1. Simulation

**Figure 3:**
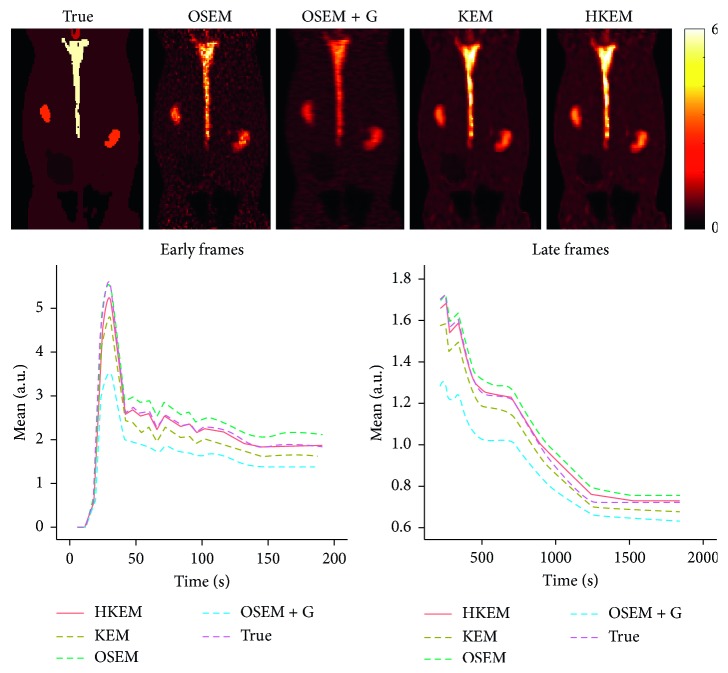
A comparison between the true and the measured IDIF values over time, as obtained from the reconstructed image with HKEM, KEM, OSEM, and OSEM+G. On the top, the peak frame (24–30 s) images are also shown.

**Figure 4:**
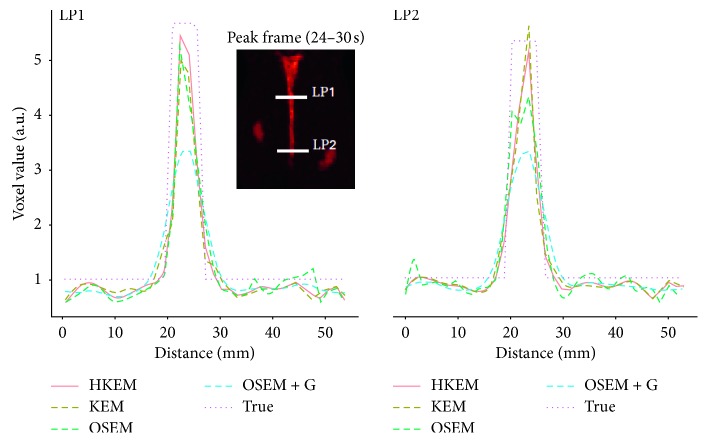
A comparison between the true line profiles, LP1 and LP2, and the ones obtained from the reconstructed image with OSEM, OSEM+G, KEM, and HKEM.

**Figure 5:**
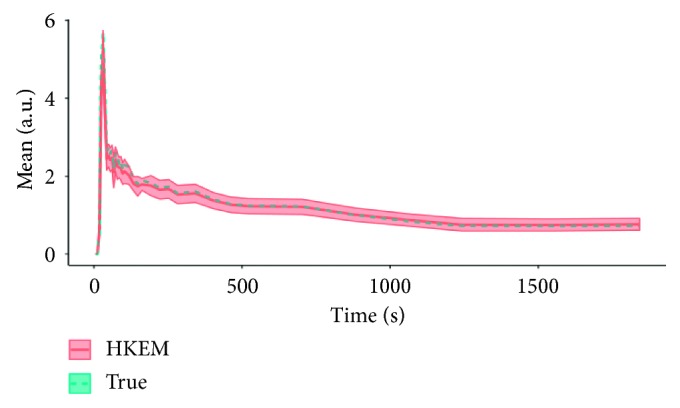
Median IDIF estimated over the ten noise realisations using the HKEM. The shaded region is the range of possible values over the 10 simulated datasets, and the dashed line is the true IDIF.

The IDIF estimates for the simulated rabbit data and the early and late frames for the IDIF are illustrated in Figure 3. In the same figure, the reconstructed images with OSEM, OSEM+G, KEM, and HKEM, at the peak frame (24–30 s), are shown.  Figure 4 presents the line profile of the aorta estimated for the images, as reconstructed with all investigated methods, at two different positions (LP1 and LP2), while Figure 5 reports the median IDIF estimated over the ten noise realisations using the HKEM. The shaded region is the range of possible values over the 10 simulated datasets, and the dashed line is the true IDIF. Finally, Table 1 reports the percentage value of the mean, maximum, and minimum absolute bias over the frames and the noise realisations.

**Figure 6:**
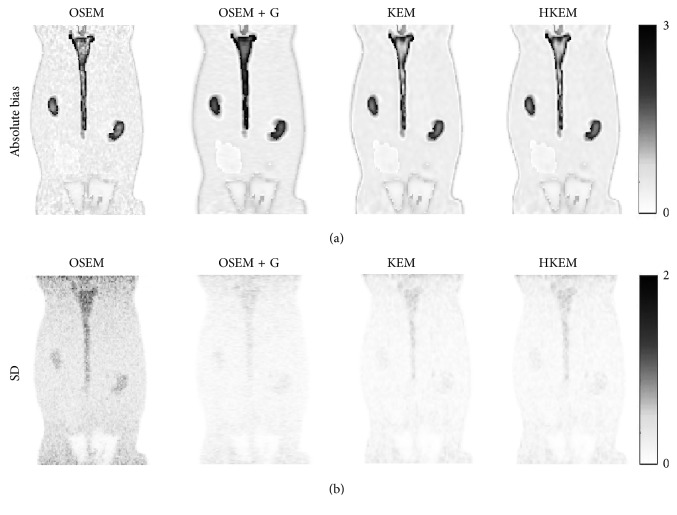
Voxel-wise image analysis over the ten noise realisations using the peak frame. The top row shows the average absolute bias, and the bottom row shows the SD over the ten noise realisations.

A voxel-wise analysis example is reported in Figure 6, where the 10 peak frame images were combined to extract the bias and the SD images for each algorithm.

### 3.2. NaF Study

**Figure 7:**
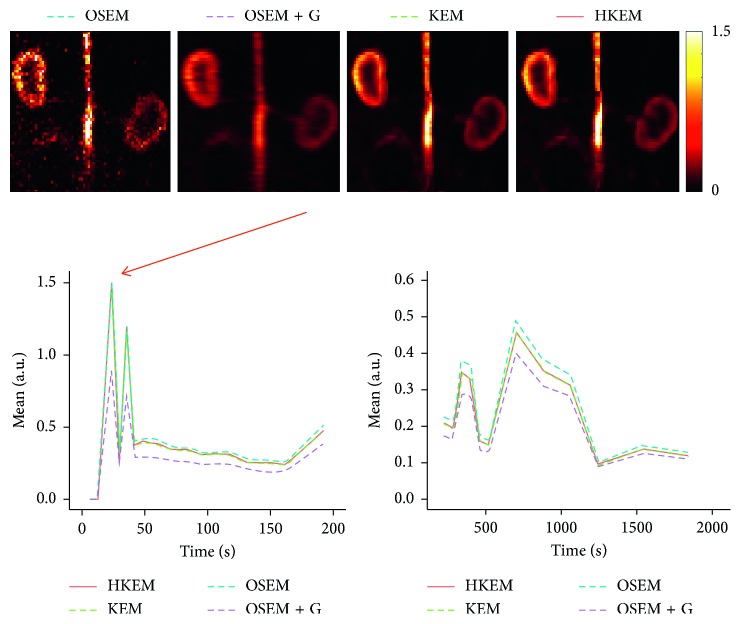
Comparison between the [^18^F]-NaF IDIF values over time, after reconstructing with OSEM, OSEM+G, KEM, and HKEM methods. On the top, the peak frame (30–36 s) images are also shown.

**Figure 8:**
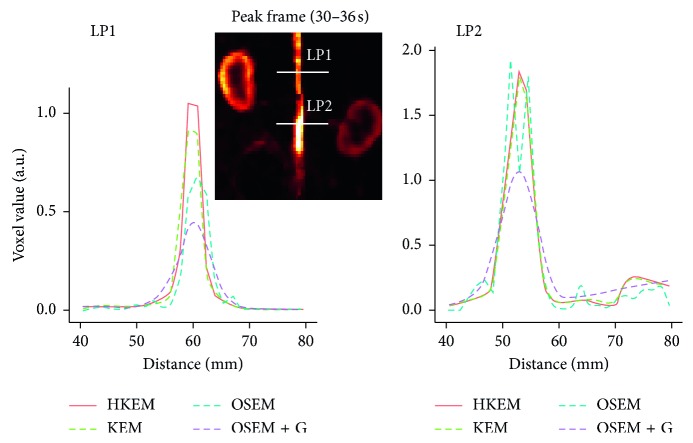
Comparison between the line profiles, LP1 and LP2, for the [^18^F]-NaF study, after reconstructing with HKEM, KEM, OSEM, and OSEM+G methods.

**Figure 9:**
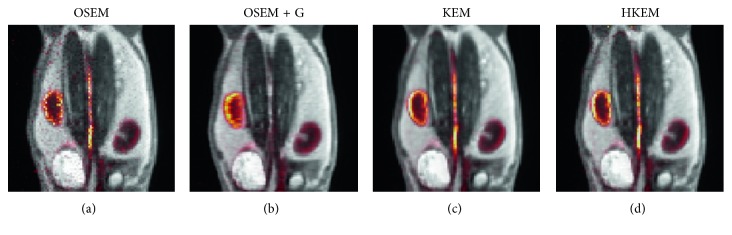
Comparison between reconstructed images with (a) OSEM, (b) OSEM+G, and (c) KEM using only MR and (d) the proposed HKEM fused with the MR UTE image for the [^18^F]-NaF rabbit data.

Figure 7 shows the comparison, on the bottom row, between the initial 200 s of the input function on the left, and the later section of the IDIF on the right. Moreover, to give an idea of the image quality, the reconstructed [^18^F]-NaF images for the peak time are shown on the top.  Figure 8 reports the line profile of the aorta in two different positions (LP1 and LP2) for the [^18^F]-NaF peak images reconstructed with the investigated methods to illustrate in detail the differences between the images reconstructed with different techniques.  Figure 9 gives an example of fused PET/MR image quality for all the reconstruction techniques.

### 3.3. FDG Study

**Figure 10:**
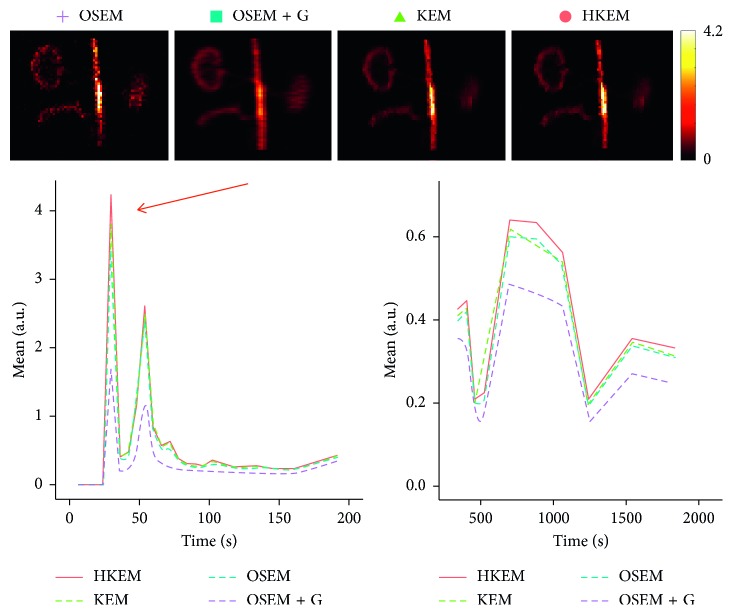
Comparison between the IDIF values over time, after reconstructing with OSEM, OSEM+G, KEM, and HKEM methods for the [^18^F]-FDG rabbit data. On the top, the peak frame (30–36 s) images are also shown.

**Figure 11:**
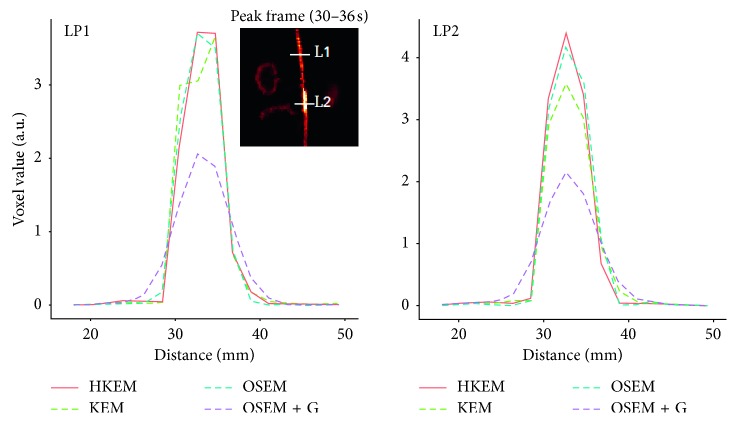
Comparison between the line profiles, LP1 and LP2, after reconstructing with OSEM, OSEM+G, KEM, and HKEM methods for the [^18^F]-FDG rabbit data.

The IDIF was estimated for a [^18^F]-FDG study in order to assess the method on a different tracer.  Figure 10 shows a comparison among the different algorithms in terms of image quality at the [^18^F]-FDG peak activity frame, input function values. On the bottom row, we can see the initial 200 s of the input function on the left and the remaining part of the IDIF on the right, while on the top, the reconstructed images for the peak frame are shown.  Figure 11 reports the line profile of the aorta in two different positions (LP1 and LP2) for the [^18^F]-FDG peak images reconstructed with all the investigated methods.

## 4. Discussion

In this study, we have proposed the use of our recently developed hybrid kernelised reconstruction algorithm HKEM, for the estimation of the IDIF in the aorta artery of rabbits having undergone [^18^F]-FDG and [^18^F]-NaF PET/MR studies using a clinical PET/MR scanner. The study was driven by the fact that many applications, where dynamic PET is used to extract more accurate and precise kinetic imaging biomarkers, rely on the estimation of the IDIF which is problematic in preclinical studies due to extensive PVE. As a consequence, it is relevant to propose a method which provides accurate estimates of IDIF. The results in Figure 3 show that the proposed reconstruction method and ROI extraction provide accurate results for all time points. The mean, maximum, and minimum bias were also calculated over the frames and the ten noise realisations (Table 1). We were able to obtain a mean bias of 5% using the HKEM with the maximum value being 16.1%. Note that due to the applied threshold in the definition of the ROI, the OSEM also provided accurate results although the dynamic PET image frames were very noisy, and thus it becomes challenging to accurately delineate the appropriate aortic input function ROI, which is crucial for the IDIF calculation. In addition, a 52% averaged CoV over noise realisations means that there is a probability of about 68% that the repeated measure will have a value within ±52% around the mean. As a consequence, values with high bias are very likely with OSEM. The results suggest that MR information can provide substantial improvement in terms of PVE and noise suppression. Nevertheless, the inclusion of the PET functional information allows better accuracy at similarly low noise levels (Table 1), compared to KEM.  Figure 4 shows the line profiles in two different points of the carotid for the image corresponding to the peak. Here, we can notice the better delineation of the aorta for both the KEM and HKEM MR-guided techniques, thanks to the broader smoothness applied in the background tissue regions. It is also important to highlight that the extraction of the ROI from the OSEM image in Figure 1 would not be accurate, as the maximum value was very high due to noise. Thus, the 75% thresholding would only extract very few voxels, therefore causing up to 100% bias in the OSEM IDIF values despite being associated with high accuracy estimates. Figure 5 illustrates the median full IDIF estimate over the 10 realisations, and it is possible to notice the accuracy over time compared to the true values. A voxel-wise analysis example is reported in Figure 6, where it can be seen the better image quality of KEM and HKEM, with lower bias in the aorta and low SD overall. The ROI analysis was also performed on this image. The results reported in Table 2 are in agreement with the ROI analysis performed with all the frames. Due to the optimized ROI, OSEM gives a similar bias value to HKEM on the peak frame; however, the repeatability of the measure is around 3 times worse. When Gaussian filter is applied, the value is extremely biased with similar CoV to HKEM and KEM.

The same analysis was applied to two real PET/MR rabbit datasets acquired with the Biograph mMR scanner, using [^18^F]-NaF or [^18^F]-FDG radiotracers. Figure 7 shows consistent results for the IDIF plots. The reconstructed images using the real data show regions of high uptake only in some places of the aorta, thereby demonstrating the benefit in contrast and resolution of exploiting a hybrid PET/MR kernel matrix.  Figure 8 presents the line profiles obtained with the different methods, showing the good resolution of the aorta when using the HKEM method and the poor quality of the postfiltered OSEM which is highly affected by the PVE. In Figure 9, the fused PET/MR image is illustrated for each technique, confirming the better alignment of the aorta region between the PET and the MR images and the resulting higher PET image resolution and aortic contrast. Moreover, the comparison between the [^18^F]-FDG and [^18^F]-NaF PET/MR studies allowed to assess the feasibility and performance of HKEM in estimating the aorta IDIF for two of the most commonly employed radiotracers in oncology and cardiology. From the results in Figure 10, the benefit of the synergistic PET/MR information encoded in the kernel matrix is visible especially in the IDIF plot. These results are also supported by the line profiles in Figure 11 showing a clear definition of the aorta for the proposed method and minimum spill-out of activity from the aorta. It is worth noticing that, for the real data, there are two peaks in the early frames IDIF; this is probably due to the fact that the injection was not continuous during the scan but there was a sudden stop making the uptake rate drop down in that specific time frame. We could show the IDIF with one peak by summing the frame associated with the first peak and the frame having low uptake; however, we think it is interesting to show the effect of a noncontinuous injection on the IDIF estimation. The input function represents a very crucial data component when estimating kinetic parameters, and its accurate estimation can become extremely challenging for small animal imaging due to the very small sizes of the associated aortic vessels. In this study, we proposed the use of PET/MR synergistic information for the more accurate and precise extraction of the aortic ROI and IDIF estimation in the framework of the HKEM method. We demonstrated that, despite the small size of the rabbit aorta, it is feasible and promising to employ the HKEM method for the extraction of an aorta IDIF estimate of improved accuracy and reduced PVE even when using a clinical PET/MR scanner. In addition, the method described to extract the ROI is easy to use and implement as it only involves trivial mathematics between matrices. It is worth mentioning that, although this study was performed with PET/MR data, it could also work with PET-CT data especially if the CT image to use as anatomical information is a CT angiography image.

## 5. Conclusion

In this investigation, we demonstrated that the HKEM method allows the more accurate extraction of the aortic ROI for improved IDIF estimation even when using a human hybrid scanner, compared to conventional OSEM or anatomically guided KEM reconstruction. Our findings were validated with both 10 simulated [^18^F]-NaF PET/MR datasets as well as 2 real rabbit PET/MR studies. Further, the methodology can be applied to most of the available radiotracers and with PET-CT without any major modification. This technique can enhance the use of dynamic PET in the context of imaging biomarkers with direct pharmacokinetic information.

## Figures and Tables

**Table 1:** Absolute bias (%) and CoV (%) estimation over the 45 frames.

	Mean bias	Max. bias	Min. bias	Mean CoV	Max. CoV	Min. CoV
OSEM	6.3	20.8	0.1	52.0	75.6	31.5
OSEM+G	23.32	39.2	4.4	16.2	34.9	10.1
KEM	12.8	30.2	7.7	19.3	30.4	10.9
HKEM	5.0	19.3	0.8	19.9	32.8	10.7

**Table 2:** Bias (%) and CoV (%) estimation over the 10 noise realisations at the peak frame.

	Bias	Mean CoV
OSEM	19.3	15.4
OSEM+G	40.4	3.5
KEM	20.2	4.3
HKEM	19.3	4.6

## Data Availability

A demonstrative code for the creation of the simulated study, reconstruction, and ROI extraction is available in CODE OCEAN at https://doi.org/10.24433/CO.bde84e0c-4c73-47fa-8ba5-81fb8bd2af77. The real rabbit data used to support the findings of this study, however, have not been made available because the Translational and Molecular Imaging Institute Group, who provided the data, retains the right to publish the data before making them generally available.
